# Progranulin Gene Delivery Protects Dopaminergic Neurons in a Mouse Model of Parkinson’s Disease

**DOI:** 10.1371/journal.pone.0097032

**Published:** 2014-05-07

**Authors:** Jackalina M. Van Kampen, David Baranowski, Denis G. Kay

**Affiliations:** 1 Neurodyn Inc., Charlottetown, Prince Edward Island, Canada; 2 Department of Biomedical Science, University of Prince Edward Island, Charlottetown, Prince Edward Island, Canada; 3 Department of Pathology and Microbiology, University of Prince Edward Island, Charlottetown, Prince Edward Island, Canada; National Institutes of Health, United States of America

## Abstract

Parkinson’s disease (PD) is a progressive neurodegenerative disorder characterized by tremor, rigidity and akinesia/bradykinesia resulting from the progressive loss of nigrostriatal dopaminergic neurons. To date, only symptomatic treatment is available for PD patients, with no effective means of slowing or stopping the progression of the disease. Progranulin (PGRN) is a 593 amino acid multifunction protein that is widely distributed throughout the CNS, localized primarily in neurons and microglia. PGRN has been demonstrated to be a potent regulator of neuroinflammation and also acts as an autocrine neurotrophic factor, important for long-term neuronal survival. Thus, enhancing PGRN expression may strengthen the cells resistance to disease. In the present study, we have used the 1-methyl-4-phenyl-1,2,3,6-tetrahydropyridine (MPTP) model of PD to investigate the possible use of PGRN gene delivery as a therapy for the prevention or treatment of PD. Viral vector delivery of the PGRN gene was an effective means of elevating PGRN expression in nigrostriatal neurons. When PGRN expression was elevated in the SN_C_, nigrostriatal neurons were protected from MPTP toxicity in mice, along with a preservation of striatal dopamine content and turnover. Further, protection of nigrostriatal neurons by PGRN gene therapy was accompanied by reductions in markers of MPTP-induced inflammation and apoptosis as well as a complete preservation of locomotor function. We conclude that PGRN gene therapy may have beneficial effects in the treatment of PD.

## Introduction

Progranulin (PGRN) is a secreted 593 amino acid multifunction protein that is highly conserved and found in a wide range of species ranging from eukaryotes to humans. PGRN is widely distributed throughout the CNS where it is found primarily in neurons and microglia but has also been detected, at much lower levels, in astrocytes and oligodendrocytes [1], [2]. PGRN first gained attention as a CNS protein of interest due to the association of *PGRN* mutations with neurodegenerative disease. Mutations in *PGRN* were first identified in association with ubiquitin-positive frontotemporal lobar degeneration linked to chromosome 17q21 (FTLDU-17) [3]–[5]. A 50% reduction of functional PGRN (haploinsufficiency) leads to increased neuronal cell death in adult FTLD brains [6]. To date, more than 113 *PGRN* mutations have been identified. Although *PGRN* mutations have been predominantly observed in FTLDU patients, the high degree of phenotypic variability associated with these mutations suggests its possible involvement in other neurodegenerative brain diseases, including Parkinson’s disease (PD) [7], [8]. Indeed, parkinsonism is displayed in some FTLD patients and is more common (up to 41%) in those patients with *PGRN* mutations [9], [10].

Recent research has implicated PGRN in embryogenesis, wound repair and inflammation, and cell growth and survival. In mice, loss of PGRN exaggerates indices of an ageing brain, dysregulates inflammatory responses, increases susceptibility to cytotoxic stresses, reduces synaptic connectivity and impairs plasticity [11]–[13]. Consistent with its strong immunoreactivity in activated microglia [3], [14], PGRN is known to be a critical regulator of CNS inflammation [11], [15], [16]. This may explain reports of upregulated expression in numerous disease states involving microglial activation, including motor neuron disease, lysosomal storage disease, and Alzheimer’s disease [3], [17]–[19]. Collectively, recent findings also suggest that PGRN functions as an autocrine neuronal growth factor, important for long-term neuronal survival [20], [21]. Thus, PGRN has the potential to influence susceptibility to a wide range of neurodegenerative diseases, including PD.

Treatment for PD remains largely symptomatic, with no effective disease-modifying therapy available for these patients. Thus, the development of interventions aimed at slowing disease progression represents a critical unmet need in the field of PD. The present study was designed to determine the neuroprotective effects of enhanced PGRN expression in a rodent model of PD. By transducing nigral neurons using the lentiviral vector, ND-602, we report on the effects of enhanced PGRN expression on neuronal survival in the MPTP mouse model of PD. We show that ND-602 effectively increases PGRN expression in neurons of the SN_C_, strengthening their resistance to MPTP-induced toxicity and preventing the development of locomotor deficits.

## Materials and Methods

### Ethics Statement

All animal experimentation was conducted in accordance with the CCAC guidelines for the care and use of laboratory animals and were approved by the University of Prince Edward Island institutional Animal Care Committee (ACC).

### Animals

All studies used male C57Bl6 mice (Charles River), approximately 3 months of age. Animals were housed in a temperature-controlled environment with a 12 h light/dark cycle and *ad libitum* access to standard chow and water. All surgery was performed under inhalable isoflurane anesthesia, and all efforts were made to minimize suffering.

### Lentiviral Construct

Progranulin-expressing lentiviral vector was designed and produced under contract by Invitrogen Corporation (Carlsbad, CA). Briefly, the viral vector was generated as such: (1) PCR amplified the PGRN gene from a plasmid provided by Neurodyn. (2) cloned PGRN into a Gateway Entry vector. Full-length sequencing of the insert to confirm the final construct. (3) performed a Gateway LR Recombination to transfer PGRN from the Gateway Entry vectors into pLenti6/R4R2/V5-DEST Expression vector. (4) performed the plasmid preparations (1L of culture) for the pLenti construct. (5) the resulting pLenti plasmid DNA was used for Lentivirus Production Service. The titer was determined to be 1×10^8^ TU/mL by the blasticidin resistance assay.

### Lentiviral Delivery

Animals received acute intranigral infusion of either ND-602, or a GFP-expressing control vector, 4 weeks prior to MPTP intoxication. Thus, animals were anaesthetized using isoflurane (1%) and placed in a Kopf stereotaxic frame. The ND-602 was injected unilaterally the left substantia nigra (A.P. 0.2.9, M.L. +1.30, D.V. −4.10) at a rate of 0.2 µl/minute via an infusion cannula connected by polyethylene tubing (50 PE) to a 50 µl Hamilton syringe driven by a Harvard pump. Following infusion, the viral vector was permitted to diffuse away from the cannula for two minutes before withdrawal.

### MPTP Intoxication

Four weeks following viral vector delivery, animals were exposed to the parkinsonism-inducing neurotoxin, 1-methyl-4-phenyl-1,2,3,6-tetrahydropyridine hydrochloride (MPTP HCl) (Sigma-Aldrich). Animals received daily injections of either MPTP-HCl (30 mg/kg, i.p.) or its vehicle, saline (0.9%) for 5 days.

### Behavioural Analysis

Two weeks following the final MPTP injection, animals underwent assessments for locomotor impairments. Locomotor activity was measured for 1 hour using a home cage video tracking system (MED Associates Inc.), designed to record total ambulatory distance, time spent ambulatory, number of ambulatory events, average velocity, time spent at rest, rotational behaviour (measuring asymmetry) and rearing. Locomotor coordination was assessed using a Foot Misplacement Apparatus (Columbus Instruments). Briefly, animals were trained for 3 days to traverse a horizontal ladder. The ladder is open on one end, with a bright light, and a dark enclosure is located on the other end. An electrified plate, located under the rungs of the ladder, serves to provide a mild electrical shock during training and to record foot slips during testing. Following 3 days of training, the total number of foot slips, along with travel time, were recorded.

### Immunohistochemistry

Immediately following behavioural testing, animals were sacrificed by transcardial perfusion with 4% paraformaldehyde. Brains were removed and postfixed for 24 hours in 4% paraformaldehyde followed by cryoprotection in 30% sucrose. Symmetrical 20 µm-thick sections were cut on a freezing microtome and stored in a Millonigs solution. Every twelfth section was processed for immunohistochemistry. Free-floating sections were incubated in 0.3% Triton X-100/Tris-buffered saline for 15 minutes, in blocking solution (3% goat serum/0.3% Triton X-100/Tris-buffered saline [TBS]) for 1 hour at room temperature, followed by the appropriate primary antibody at 4°C overnight. Primary antibodies included tyrosine hydroxylase (TH) (Chemicon MAB 318, 1∶10,000), dopamine transporter (DAT) (Chemicon MAB 369, 1∶1000), GFP (Chemicon AB16901, 1∶600), PGRN (R&D Systems, 1∶1000), and activated caspase-3 immunolabeling (AbCam AB2302, 1∶500). For fluorescent visualization, sections were incubated with the respective secondary antibody conjugated to either Alexa 488, Alexa 594, or Alexa 350 (Molecular Probes). In between steps, sections were washed for 3×10 minutes in TBS. Sections were mounted on unsubbed glass slides and coverslipped in Fluoromount. Sections were also assayed for inflammation using fluorescein-conjugated isolectin B_4_ (ILB4) (Molecular Probes, 10 µg/ml). Nissl was assessed in free-floating sections using NeuroTrace (Invitrogen, 1∶50). For the *in situ* terminal deoxynucleotidyl transferease dUTP nick end labeling (TUNEL) assay (R&D Systems), sections were first mounted on clean glass slides and processed according to the manufacturer instructions. Fluorescence signals were detected with a Zeiss AxioObserver Z1 Imaging microscope equipped with an apotome system at excitation/emission wavelengths of 535/565 nm, 470/505 nm, and 585/615 nm.

### Image Analysis

Nigrostriatal integrity was determined by TH^+^ cell counts in the SN_C_ and striatal TH and DAT immunodensity. As TH^+^ cell counts are only a marker of dopaminergic phenotype and do not necessarily imply cell death/survival, lesion severity was determined by assessment of both TH^+^ and Nissl^+^ cell counts. Stereological analyses of TH-positive and Nissl-positive neurons in the SNc were carried out by a blind experimenter using the optical fractionator method, an unbiased method of cell counting that is independent of the volume of the brain area considered and the size of neurons being counted. The SNc was delineated at 10x magnification using the Paxinos and Watson atlas for anatomical landmarks. TH^+^ neurons were counted from every 6^th^ serial section (20 µm) using a Zeiss AxioObserver Z1 Imaging microscope equipped with an apotome system for z stacking, area estimation and 3D reconstruction. The microscope was interfaced to a Dell Dimension 8100 workstation with Axiovision software. A counting frame of x = 100 µm and y = 100 µm was incorporated along with steps involving movements of x = 140 µm and y = 140 µm. For denstiometric analyses of striatal TH or DAT, a sampling frame of x = 50 mm, y = 50 mm, and z = 12 mm was used to sample the dorsomedial, dorsolateral and ventrolateral regions of each of 4 sections through the striatum using Axiovision software. Pyknotic cell nuclei in Nissl-stained sections were used as a morphological measure to estimate the occurrence of cell death. Pyknotic cells were counted in every 12^th^ section, across 4 sections through the SN_C_. Cells were considered pyknotic if they lacked a nuclear membrane, had pale or absent cytoplasm and darkly stained condensed chromatin. We also examined the density of PGRN immunoreactivity in the SN_C_. In 4 sections, the SN_C_ was identified by TH immunolabeling and outlined. Densitometric measurements of the enclosed area were obtained using Axiovision software (Zeiss). In addition, we also examined the density of PGRN immunolabeling within TH^+^ neurons. To do this, we located a TH^+^ neuron, imaged it in 3 dimensions, and obtained densitometric measurements from the center 1.5 µm layer of the cell using a 5 µm×5 µm sampling frame. Ten samples were obtained for each of 4 serial sections through the SN. Where applicable, double-labeling was confirmed by rotating the image along each axis to ensure signals were localized within the same cell rather than separate cells in close apposition.

#### HPLC

Following behavioural testing, a subset of animals were sacrificed by cervical dislocation and the brains were removed. The striatum was dissected, immediately frozen on dry ice, and stored at −80°C before assays were performed. Tissues were weighed, ultrasonicated in 10% perchloric acid, and centrifuged at 20,000*×g* for 10 min. The levels of DA and its metabolites, 3,4-dihydroxyphenylacetic acid (DOPAC), and homovanillic acid (HVA), in brain tissue extracts were determined by HPLC coupled with an electrochemical detector. The supernatant was removed and filtered by centrifugation using Nanosep (300MWCO) microcentrifuge vials at 10,000 RPM at 4°C for 60 minutes. The filtrate was diluted 1∶2 in 0.4 M HClO_4_ and analyzed by LC/MS/MS. Supernatant aliquots (20 µL) were then injected into an HPLC equipped with a 3 µm C18 column. The mobile phase was comprised of 26 mL of acetonitrile, 21 mL of tetrahydrofuran, and 960 mL of 0.15 M monochloroacetic acid (pH 3.0) containing 50 mg/L of EDTA and 200 mg/mL of sodium octyl sulfate. The amounts of DA, DOPAC, and HVA were determined by comparison of peak areas of tissue samples with authentic standards, and were expressed in µg/g of wet tissue. Protein content was determined using a Pierce BCA Protein Assay Kit (Thermo Scientific, 23227) as per manufacturer’s instructions using the Microplate Procedure. Some samples were also analyzed for the MPTP metabolite, MPP^+^.

The HPLC system consisted of Shimadzu components: System controller (SCL 10A VP) Liquid Chromatograph (LC-10A VP), Eletrochemical Detector (L-ECD-6A), Column Oven (CTO-10A), Automatic Sample Injector (SIL-10AF), Mixer (FCV-10A1 VP) and Degasser (DGU-14A). All components were integrated using a Class VP (version 7.2 SP1 Rev. B) software.

A Gemini NX 5u, C18, 250×4.6 mm column (Phenomenex, OOG-4454-EO) was kept in the colum oven where the temperature was maintained at 28 C. The mobile phase was 11.5% methanol (or 10% methanol, depending on peak separation) and Rezvani mobile phase (Rezvani et al., 2008). The mobile phase was continually degassed and delivered at a flow rate of 1.0 mL/min. Each 50 uL injection lasted 27 minutes at 700 mV with an average pressure of ∼1600 psi.

### Data Analysis

Data were analyzed using a multivariant analysis of variance. Where significant F-values were obtained, planned pair-wise comparisons were made using Newman-Keuls. Differences were considered statistically significant when *p*<0.05.

## Results

### Intranigral Infusion of ND-602 Increases PGRN Expression in the SN_C_

**Figure 1:**
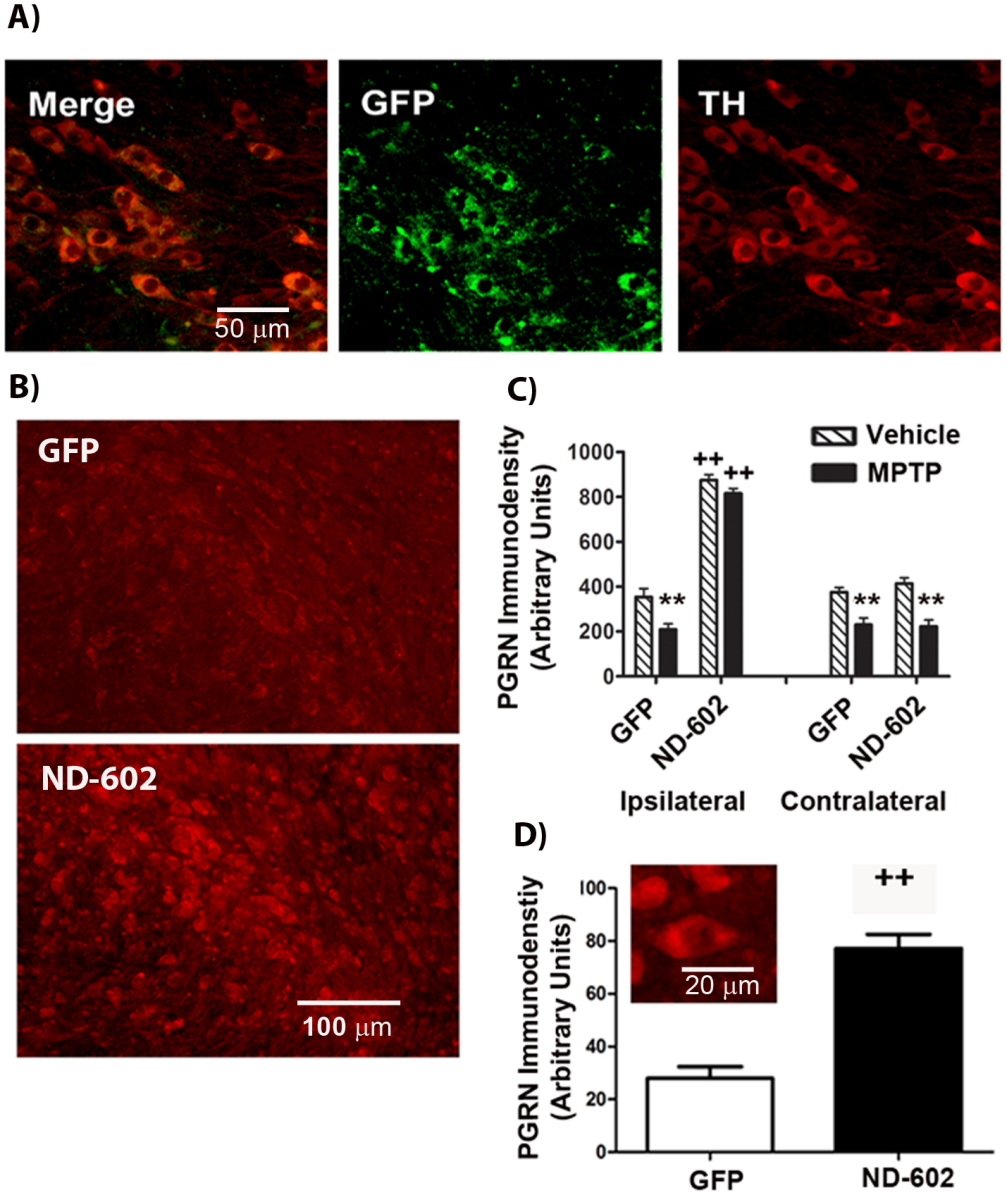
Immunohistochemical analysis of enhanced PGRN expression in the SN_C_ following ND-602. **A)** Fluorescent photomicrographs depicting GFP expression in TH^+^ neurons of the ipsilateral SN_C_ 4 weeks following unilateral intranigral ND-602 infusion. **B)** Fluorescent photomicrographs depicting PGRN immunolabeling in the ipsilateral SN_C_ 4 weeks following unilateral intranigral infusion of either ND-602 or GFP control. **C)** In GFP controls, MPTP produced a significant reduction in PGRN immunolabeling within the substantia nigra of both hemispheres. Unilateral intranigral delivery of ND-602 significantly elevated PGRN immunolabeling in the ipsilateral SN_C_ of both vehicle- and MPTP-treated animals. No effect of ND-602 was seen in the contralateral nigra. Each bar represents the mean (±S.E.M., *n* = 5–9) optical density (arbitrary units) averaged across 4 coronal sections through the SN_C_. **D)** The density of PGRN immunolabeling within TH^+^ neurons of the ipsilateral SN_C_ was significantly elevated in those animals treated with ND-602. Inset fluorescent photomicrograph depicts PGRN immunolabeling in dopaminergic neurons of the ipsilateral SN_C_ following ND-602 treatment. Each bar represents the mean (±S.E.M., *n* = 5–9) optical density (arbitrary units) measured in 10 cells in each of 4 coronal sections through the ipsilateral SN_C_. **sig. diff. from vehicle control, *p*<0.001. ++sig. diff. from GFP control, *p*<0.001.

In order to assess the efficacy of this gene delivery approach, we examined immunolabeling for the reporter protein, green fluorescent protein (GFP), in the substantia nigra (SN_C_) of male C57Bl6 mice (3 months) approximately 4 weeks following unilateral intranigral infusion of the lentiviral construct, ND-602. We found that *in vivo* gene transfer by ND-602 resulted in detectable GFP expression in 63% (±16.78) of tyrosine hydroxylase (TH)-positive neurons in the ipsilateral SN_C_ (Figure 1a). Thus, ND-602 is capable of effectively transfecting nigral neurons. In order to confirm the induction of PGRN expression by ND-602, we also examined PGRN immunolabeling in the SN_C_ and found the density of PGRN immunolabeling to be significantly elevated ipsilateral to the site of ND-602 infusion, as compared to that seen following lentiviral delivery of GFP alone (Figure 1b, c). PGRN immunolabeling in the contralateral SN_C_ remained unchanged. The density of PGRN immunolabeling within TH^+^ cells of the ipsilateral SN was also significantly elevated following viral vector delivery of PGRN by ND-602, as compared to GFP alone (Figure 1d). These results indicate that ND-602 effectively transfects nigral cells to induce the expression of PGRN within dopaminergic neurons of the SN_C_.

### ND-602 Prevents Locomotor Deficits Resulting from MPTP Toxicity

**Figure 2:**
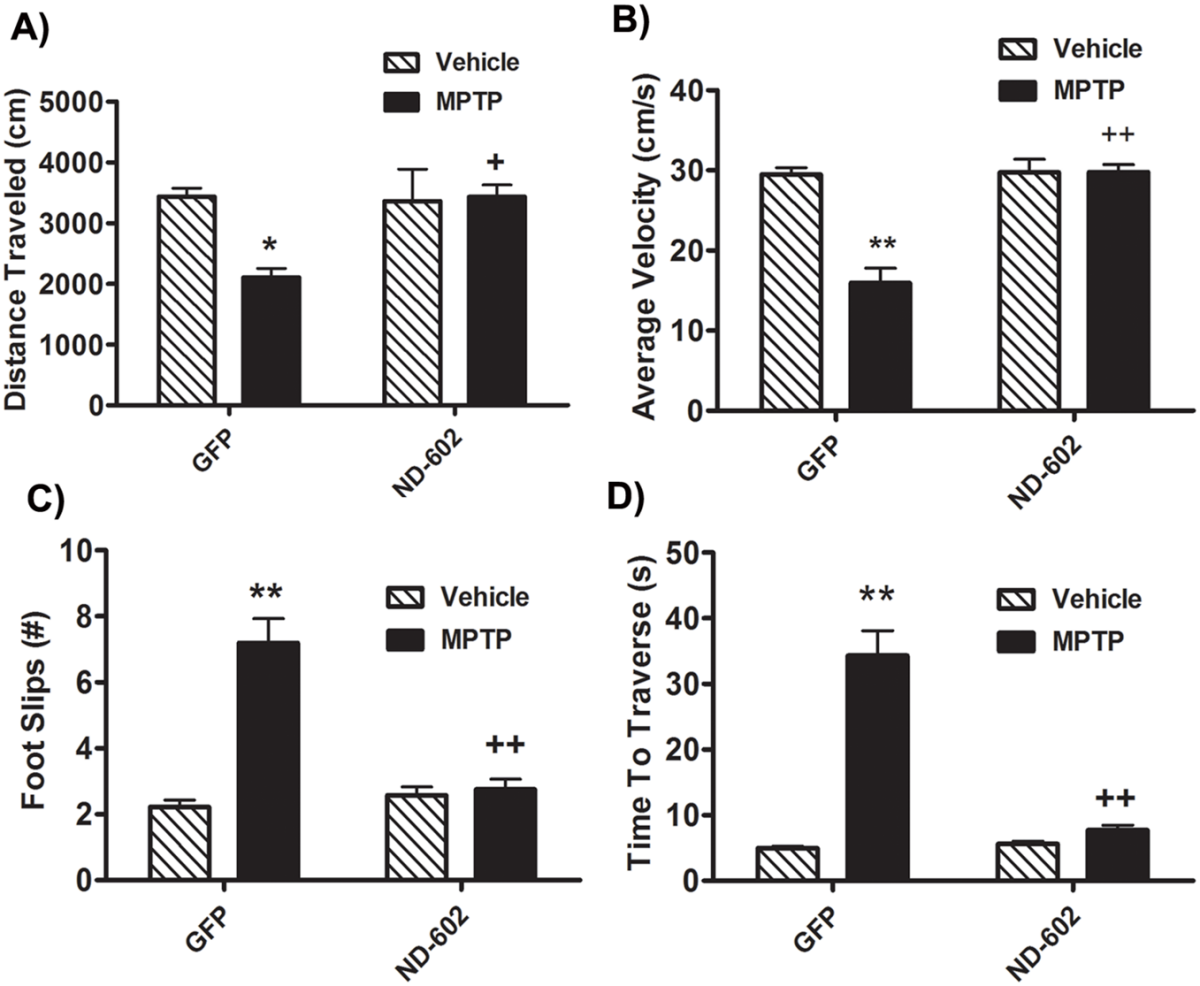
Locomotor deficits in MPTP-intoxicated mice treated with ND-602. **A)** Following MPTP intoxication, animals displayed significantly reduced locomotor activity, as assessed by total distance traveled in one hour. However, MPTP failed to reduce locomotor activity in those animals treated with ND-602. Each bar represents the mean (±S.E.M., n = 5–6) distance traveled (cm) in one hour. **B)** The average velocity of locomotion was significantly reduced following MPTP intoxication. However, in those animals treated with viral ND-602, no such reduction was observed. Velocity in those animals was significantly higher than in their GFP-treated counterparts. Each bar represents the mean (±S.E.M., n = 5–6) velocity (cm/s) observed over a one hour period. **C)** When traversing the horizontal ladder, MPTP-treated animals displayed reduced locomotor coordination as evidenced by significantly more foot slips. However, in those animals treated with ND-602, no such elevation in foot slips was observed. Locomotor coordination in those animals was significantly better than in their GFP-treated counterparts. Each bar represents the mean (±S.E.M., n = 5–6) number of foot slips recorded. **D)** Following MPTP intoxication, animals took significantly longer to traverse a horizontal ladder. However, those animals treated with ND-602 showed no increase in travel time and were significantly faster than their GFP-treated counterparts. Each bar represents the mean (±S.E.M., n = 5–6) time(s) to traverse a horizontal ladder. **sig. diff. from vehicle control, *p*<0.001; *p<0.05. ++sig. diff. from GFP control, *p*<0.001; +*p*<0.05.

Four weeks following unilateral intranigral infusion of lentiviral vector, animals were administered daily injections of the parkinsonsism-inducing neurotoxin, 1-methyl-4-phenyl-1,2,3,6-tetrahydropyridine hydrochloride (MPTP HCl) for 5 days. Two weeks following the final injection, animals underwent assessments for locomotor impairments. Locomotor activity was measured for one hour using a home cage video tracking system (MED Associates Inc.). Using this measure of akinesia/bradykinesia, MPTP was found to significantly reduce locomotor activity, as compared to saline-treated controls (Figure 2a) (MPTP main effect, F_1,21_ = 5.10193, *p* = 0.0365). However, MPTP failed to reduce locomotor activity in those animals treated with ND-602 (PGRN main effect, F_1,18_ = 5.01184, *p* = 0.038; MPTP × PGRN interaction effect, F_1,18_ = 6.32527, *p* = 0.0216). Similarly, the average velocity of locomotion was significantly reduced following MPTP treatment (Figure 2b) (MPTP main effect, F_1,18_ = 27.00992, *p*<0.0001). However, in those animals treated with viral vector delivery of PGRN by ND-602, no such reduction was observed (PGRN main effect, F_1,18_ = 29.30551, *p*<0.0001; MPTP × PGRN interaction effect, F_1,18_ = 27.20722, *p*<0.0001). Velocity in those animals was significantly higher than in their GFP-treated counterparts. Animals were also assessed for locomotor coordination using the Foot Misplacement Apparatus (Columbus Instruments). Briefly, animals were trained to traverse a horizontal ladder. Following 3 days of training, animals were given three trials and the average number of foot slips (errors) was automatically recorded. The time it took the animals to traverse the ladder was also recorded. Animals exposed to MPTP displayed significantly more errors when traversing the ladder than saline-treated controls (Figure 2c) (MPTP main effect, F_1,18_ = 9.98543, *p* = .0054). In those animals treated with ND-602, however, locomotor coordination remained intact, with no significant increase in foot slips observed in those animals (PGRN main effect, F_1,18_ = 9.98543, *p* = .0054; MPTP × PGRN interaction effect, F_1,18_ = 6.18835, *p* = .0229). MPTP-treated animals also appeared to take longer to traverse the ladder than saline-treated controls (Figure 2d) (MPTP main effect, F_1,18_ = 28.20261, *p*<0.0001) unless they had been transfected with ND-602 (PGRN main effect, F_1,18_ = 9.43567, *p* = .0066; MPTP × PGRN interaction effect, F_1,18_ = 883.23068, *p* = .0016). These results indicate that intranigral infusion of ND-602 is capable of preserving locomotor function normally impaired by MPTP intoxication.

### ND-602 Protects Nigrostriatal Neurons against MPTP Toxicity

**Figure 3:**
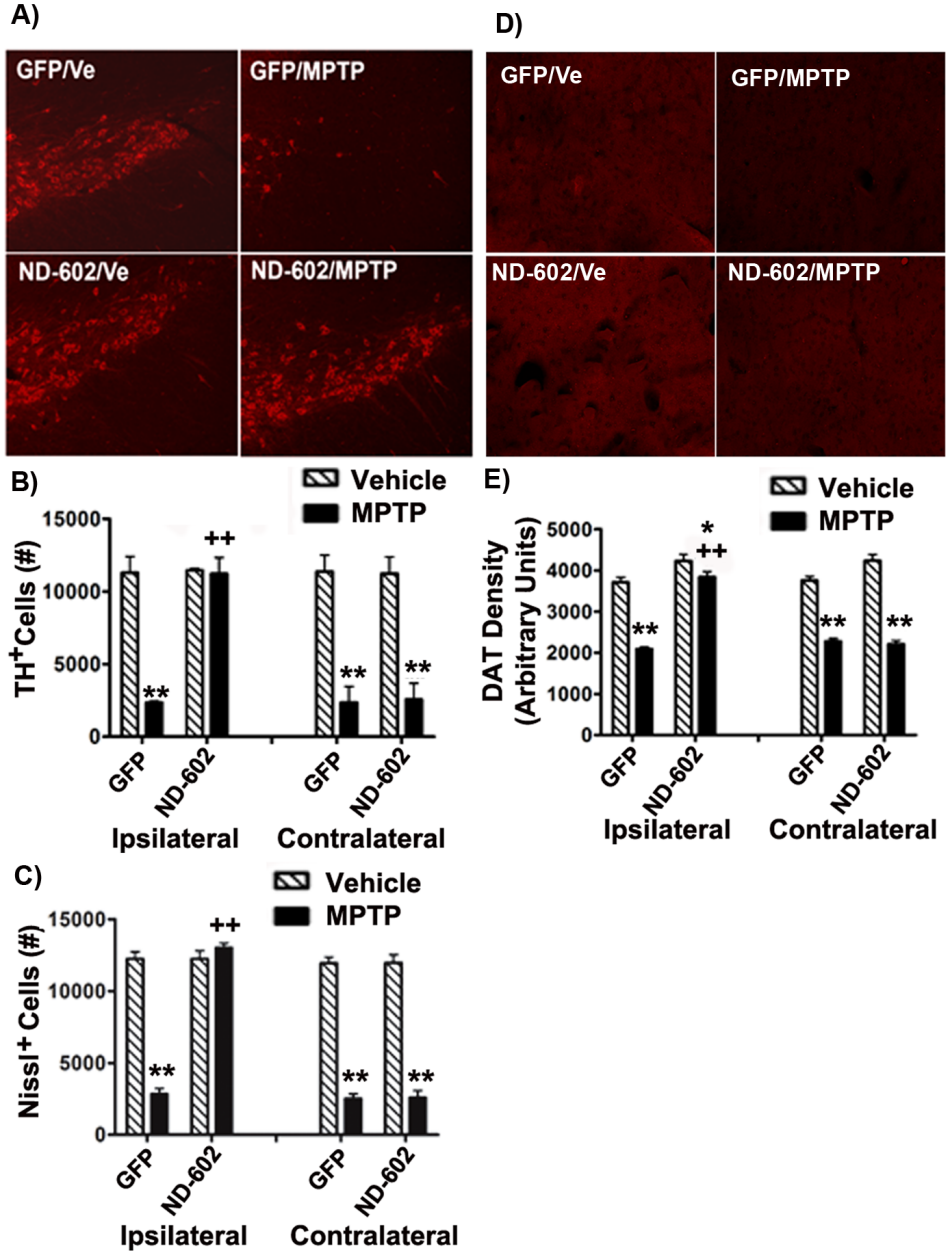
Nigrostriatal integrity of MPTP-intoxicated mice treated with ND-602. **A)** Representative fluorescent photomicrographs depicting TH immunolabeling in the ipsilateral SN_C_ following MPTP intoxication in ND-602- and GFP-treated animals. Unbiased stereological counts of **B)** TH^+^ and **C)** Nissl^+^ cells in the SN_C_ were significantly reduced following MPTP intoxication. However, no significant cell loss was observed ipsilateral to viral vector delivery of PGRN by ND-602. Significantly more TH^+^ and Nissl^+^ cells were noted ipsilateral to ND-602 infusion relative to GFP controls. Each bar represents the mean (±S.E.M., n = 5–9) number of TH or Nissl immunopositive cells counted in the SN_C_. **D)** Representative fluorescent photomicrographs depicting DAT immunolabeling in the ipsilateral striatum following MPTP intoxication in ND-602- and GFP-treated animals. **E)** MPTP intoxication resulted in a significant loss of DAT immunolabeling in the striatum. In those animals treated with ND-602, the loss of striatal DAT was significantly attenuated in the ipsilateral hemisphere. Each bar represents the mean (±S.E.M., n = 5–9) optical density (arbitrary units) measured in three striatal regions across 4 coronal sections through the striatum. **sig. diff. from vehicle control, *p*<0.001; *p<0.05. ++sig. diff. from GFP control, *p*<0.001.

Following behavioural testing, animals were sacrificed by perfusion, the brains removed and sectioned using a freezing microtome (20 µm). In order to determine nigro-striatal integrity, sections were assessed for TH and dopamine transporter (DAT) immunolabeling in the SN and striatum. As TH^+^ cell counts are only a marker of dopaminergic phenotype and do not necessarily imply cell death/survival, lesion severity was determined by assessment of both TH^+^ and Nissl cell counts. Stereological analyses of TH^+^ and Nissl^+^ neurons in the SNc revealed a significant loss of both following MPTP intoxication (Figure 3a, b, c) [TH: (MPTP main effect, F_1,27_ = 73.44052, *p*<0.0001) Nissl: (MPTP main effect, F_1,27_ = 73.72219, *p*<0.0001)]. However, in those animals treated with viral vector delivery of PGRN by ND-602, significant TH^+^ and Nissl^+^ cell loss was restricted to the untreated hemisphere, with no significant loss observed ipsilateral to the ND-602 [TH: (PGRN main effect, F_1,27_ = 1.43823, *p* = .0.2408; MPTP × PGRN interaction effect, F_1,27_ = 10.60095, *p* = .003; HEMISPHERE main effect, F_1,31_ = 14.37977, *p* = .0008; MPTP × PGRN × HEMISPHERE interaction effect, F_1,31_ = 7.55071, *p* = .0106), Nissl: (PGRN main effect, F_1,27_ = 2.9122, *p* = .0.266; MPTP × PGRN interaction effect, F_1,27_ = 58.3369, *p* = .026; HEMISPHERE main effect, F_1,31_ = 17.3407, *p* = .0005; MPTP × PGRN × HEMISPHERE interaction effect, F_1,31_ = 9.3113, *p* = .009)]. Striatal innervation by nigral neurons was determined by densitometric measurement of DAT immunolabeling in the striatum. DAT immunolabeling was reduced in the striatum following exposure to MPTP in GFP controls but not in the ipsilateral hemisphere of those animals treated with ND-602 (Figure 3d, e) [(MPTP main effect, F_1,27_ = 128.83298, *p*<0.0001; PGRN main effect, F_1,27_ = 14.05932, *p* = .0009; MPTP × PGRN interaction effect, F_1,27_ = 12.18609, *p* = .0017; HEMISPHERE main effect, F_1,31_ = 6.38948, *p* = .00176; MPTP × PGRN × HEMISPHERE interaction effect, F_1,31_ = 16.46064, *p* = .0004)]. These findings indicate that ND-602 protects against MPTP-induced nigrostriatal cell loss.

### ND-602 Preserves Striatal DA Levels following MPTP Intoxication

**Figure 4:**
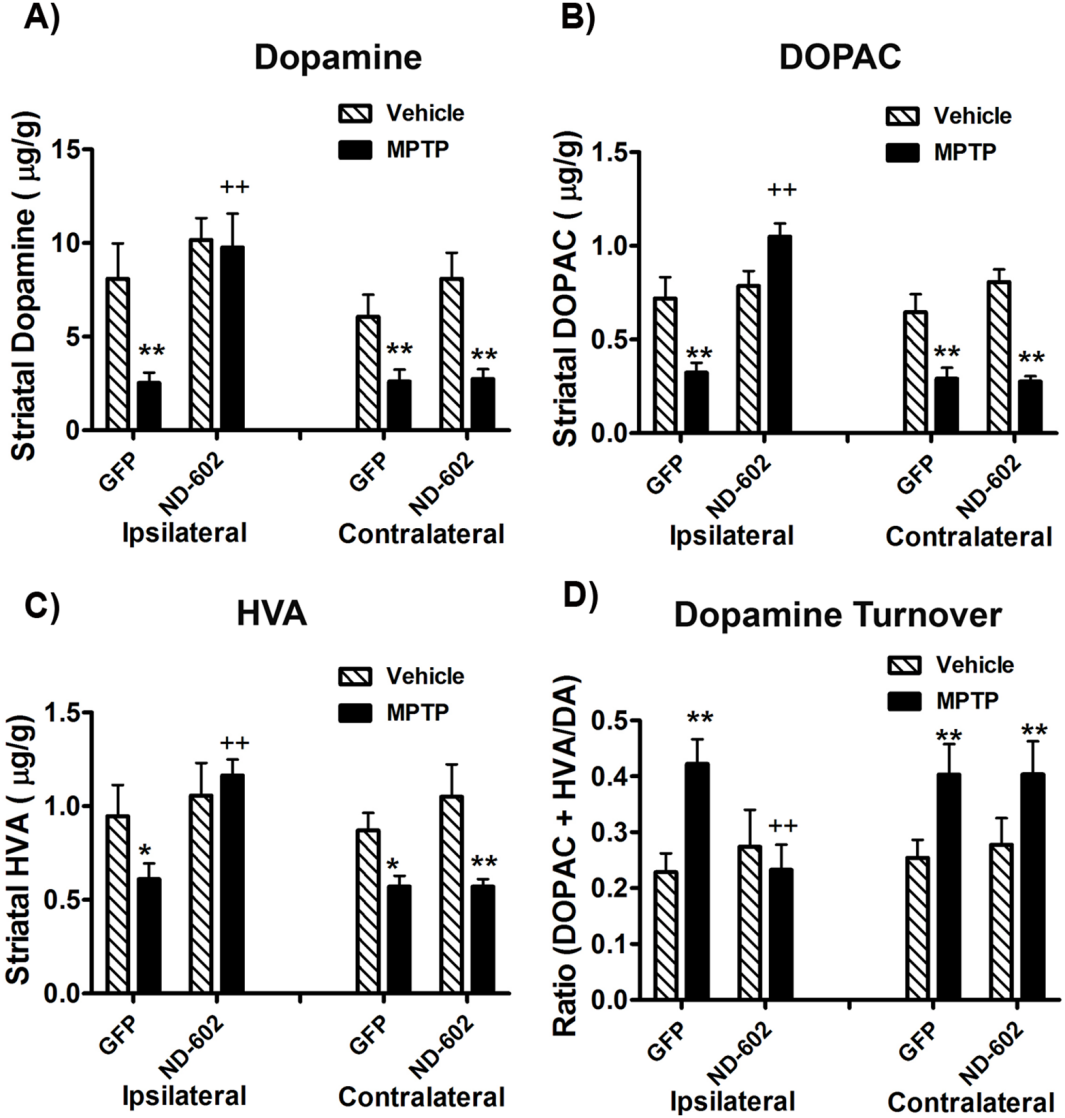
Striatal dopamine and turnover in MPTP-intoxicated mice following ND-602. **A)** Levels of striatal dopamine were significantly reduced following MPTP intoxication. However, in those animals treated with viral vector delivery of PGRN by ND-602, no decline in dopamine was observed ipsilateral to vector delivery. Levels were reduced, however, in the contralateral, untreated, hemisphere. The dopamine metabolites, **B)** DOPAC and **C)** HVA, were similarly reduced by MPTP, with no reductions observed ipsilateral to ND-602 delivery. **D)** Striatal dopamine turnover was significantly elevated by MPTP intoxication. However, no change in turnover was observed ipsilateral to ND-602 treatment. Each bar represents the mean (±S.E.M., n = 5–8) µg of dopamine, DOPAC or HVA detected per gram of wet tissue weight. For dopamine turnover, the calculation, DOPAC + HVA/dopamine was applied. **sig. diff. from vehicle control, *p*<0.001. ++sig. diff. from GFP control, *p*<0.001.

Using HPLC analysis, we found that MPTP intoxication resulted in a significant reduction in striatal DA levels (MPTP main effect, F_1,24_ = 18.32159, *p* = .0003), compared to saline-treated controls, along with the metabolites, DOPAC (MPTP main effect, F_1,24_ = 23.4348, *p*<.0001) and HVA (MPTP main effect, F_1,24_ = 10.07927, *p* = .0048) (Figure 4). In those animals treated with ND-602, however, no reduction in DA (PGRN main effect, F_1,24_ = 10.8563, *p* = .0035; MPTP × PGRN interaction effect, F_1,24_ = 0.69131, *p* = .4151; HEMISPHERE main effect, F_1,25_ = 22.98846, *p*<.0001; MPTP × PGRN × HEMISPHERE interaction effect, F_1,25_ = 9.53918, *p* = .0056) or its metabolites [HVA: (PGRN main effect, F_1,24_ = 10.82617, *p* = .0037; MPTP × PGRN interaction effect, F_1,24_ = 0.00327, *p* = .955; HEMISPHERE main effect, F_1,25_ = 13.56624, *p* = .0015; MPTP × PGRN × HEMISPHERE interaction effect, F_1,25_ = 18.09374, *p* = .0004); DOPAC: (PGRN main effect, F_1,24_ = 19.6672, *p* = .0002; MPTP × PGRN interaction effect, F_1,24_ = 4.60447, *p* = .0437; HEMISPHERE main effect, F_1,25_ = 52.09693, *p*<.0001; MPTP × PGRN × HEMISPHERE interaction effect, F_1,25_ = 49.40282, *p*<.0001)] was observed in the ipsilateral striatum. For dopamine turnover, the calculation, DOPAC + HVA/dopamine was applied. Using this calculation, dopamine turnover was determined to be elevated in the striatum of MPTP-treated animals (MPTP main effect, F_1,24_ = 5.30213, *p* = .0316), with no elevation in turnover detected ipsilateral to ND-602 treatment (PGRN main effect, F_1,24_ = 0.20854, *p* = .6526; MPTP × PGRN interaction effect, F_1,24_ = 1.67146, *p* = .2101; HEMISPHERE main effect, F_1,25_ = 19.95363, *p* = .0002; MPTP × PGRN × HEMISPHERE interaction effect, F_1,25_ = 30.9276, *p*<.0001) (Figure 4d). Thus, ND-602 not only preserves nigrostriatal dopaminergic neurons, but also prevents the loss of striatal dopamine thought to underlie alterations in locomotor function associated with this model of PD.

### ND-602 Reduces Apoptosis

To measure apoptosis in the SN_C_ of these animals, we used three different measures. First, we performed a standard terminal deoxynucleotidyl transferease dUTP nick end labeling (TUNEL) assay for detecting DNA fragmentation by labeling the terminal end of nucleic acids. Using this assay, we found that the total number of TUNEL-positive cells counted in the SN_C_ was significantly elevated following MPTP intoxication (MPTP main effect, F_1,24_ = 73.43445, *p*<.0001). However, no such elevation was observed ipsilateral to ND-602 treatment (PGRN main effect, F_1,24_ = 1.06208, *p* = .3145; MPTP × PGRN interaction effect, F_1,24_ = 1.45785, *p* = .2407; HEMISPHERE main effect, F_1,25_ = 33.88776, *p*<.0001; MPTP × PGRN × HEMISPHERE interaction effect, F_1,25_ = 16.70133, *p* = .0005) (Table 1). TUNEL labeling in the SN_C_ ipsilateral to ND-602 treatment was significantly lower than that observed in GFP controls. We also performed immunolabeling for activated caspase-3, which plays a central role in the execution phase of cell apoptosis. Here, we used an antibody which preferentially recognizes the p17 fragment of active caspase-3. Intoxication with MPTP significantly elevates the percentage of nigral neurons immunolabeled for activated caspase-3, with no such elevation observed ipsilateral to ND-602 treatment (MPTP main effect, F_1,24_ = 750.44598, *p*<.0001; PGRN main effect, F_1,24_ = 56.10479, *p*<.0001; MPTP × PGRN interaction effect, F_1,24_ = 61.62579, *p*<.0001; HEMISPHERE main effect, F_1,25_ = 20.45768, *p* = .0002; MPTP × PGRN × HEMISPHERE interaction effect, F_1,25_ = 19.75945, *p* = .0002). Finally, we also used a morphological measure of cell death by determining the percentage of Nissl-positive cells displaying a pyknotic phenotype. Cells were considered pyknotic if they lacked a nuclear membrane, had pale or absent cytoplasm and darkly stained condensed chromatin. In the SN_C_, MPTP intoxication resulted in a significant elevation in the percentage of cells displaying a pyknotic phenotype (MPTP main effect, F_1,24_ = 162.6619, *p*<.0001). However, in those animals having received viral vector delivery of PGRN by ND-602, this elevation was significantly reduced (PGRN main effect, F_1,24_ = 11.64223, *p* = .0026; MPTP × PGRN interaction effect, F_1,24_ = 8.10725, *p* = .0096; HEMISPHERE main effect, F_1,25_ = 8.46708, *p* = .0084; MPTP × PGRN × HEMISPHERE interaction effect, F_1,25_ = 4.84177, *p* = .0391). These data indicate that ND-602 may prevent nigrostriatal cell loss, in this model of PD, by reducing apoptosis.

**Table 1:** Indices of apoptosis in MPTP-intoxicated mice treated with ND-602.

	Ipsilateral				Contralateral			
	GFP		ND-602		GFP		ND-602	
	*vehicle*	*MPTP*	*vehicle*	*MPTP*	*vehicle*	*MPTP*	*vehicle*	*MPTP*
**activated** **caspase-3**	11±2.42	71.55±2.77**	9.97±1.722	23.49±2.38*^,^ ++	11.24±2.43	68.40±3.18**	13.47±2.87	65.22±5.08**
**TUNEL**	25.24±2.72	145.83±16.64**	31.25±2.57	38.54±12.25++	35.94±7.55	176.80±27.65**	34.66±9.36	224.24±24.54**
**pyknosis**	4.91±0.86	34.03±3.41**	3.42±0.35	14.62±2.50** ^,^ ++	5.39±1.09	35.01±3.56**	4.76±0.82	30.85±3.14**

Three indices of apoptosis were measured in the SN_C_, activated caspase-3 immunolabeling, TUNEL labeling, and pyknotic morphology. All three indications were significantly elevated following MPTP intoxication. However, MPTP-induced apoptosis was significantly reduced ipsilateral to viral vector delivery of PGRN by ND-602, while remaining unchanged in the contralateral hemisphere. Each number represents the mean (±S.E.M., n = 5–9) number of positive cells counted across 4 coronal sections through the SN_C_.

**sig. diff. from vehicle control, *p*<0.001; *p<0.05.

++ sig. diff. from GFP control, *p*<0.001.

### ND-602 Reduces Inflammation

**Figure 5:**
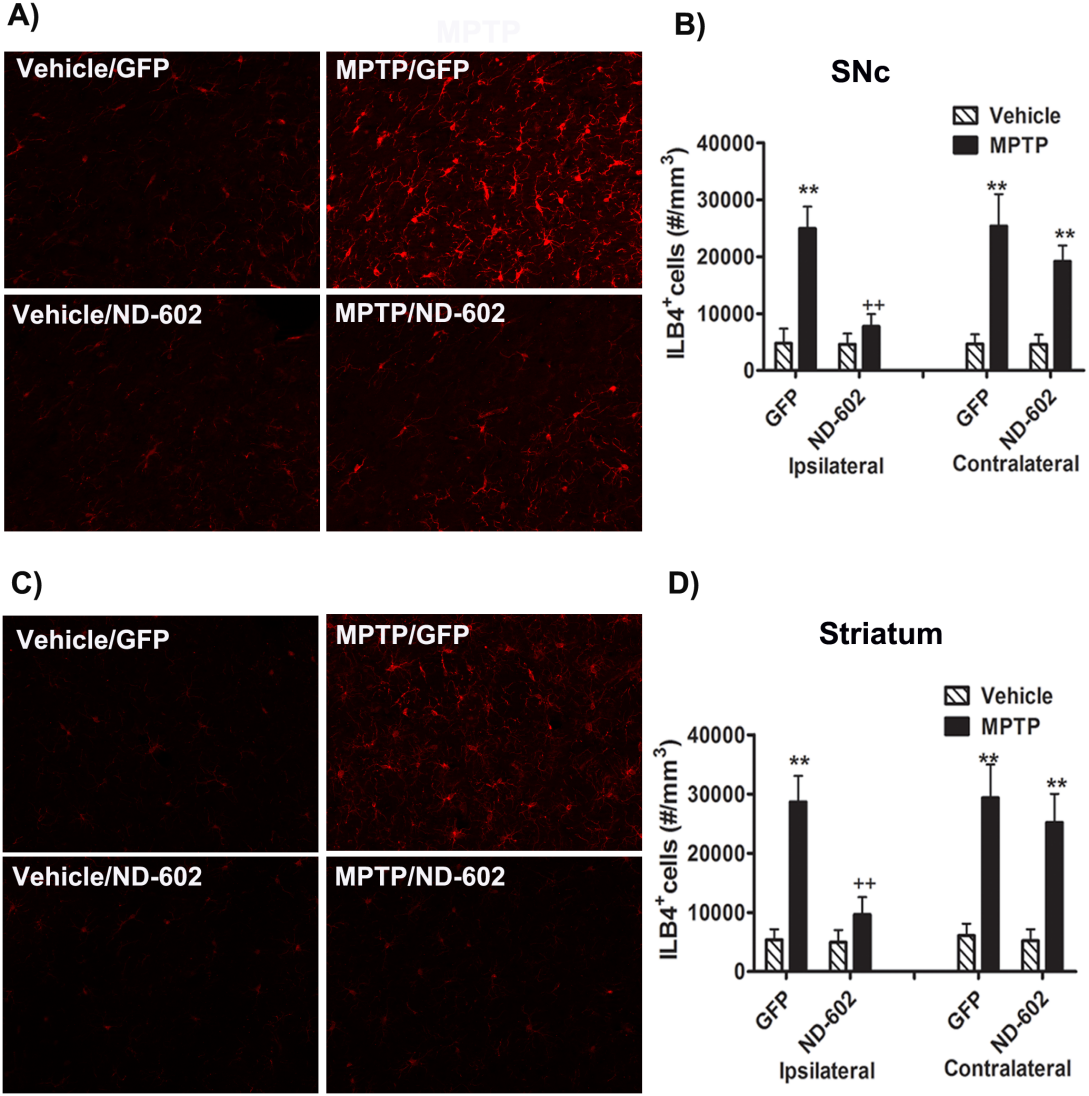
Nigrostriatal inflammation in MPTP-intoxicated mice treated with ND-602. Representative fluorescent photomicrographs of isolectin (ILB4) labeling in the **A)** SN and **C)** striatum two weeks following the last MPTP injection. MPTP intoxication resulted in a significant elevation in the number of activated microglia in both the **B)** SN_C_ and **D)** striatum. In both regions, this activation was significantly reduced in those animals treated with ND-602. Each bar represents the mean (±S.E.M., n = 5–6) number of ILB4-positive cells estimated per mm^3^. **sig. diff. from vehicle control, *p*<0.001; *p<0.05. ++sig. diff. from GFP control, *p*<0.001.

Neuroinflammatory mechanisms are believed to contribute to the cascade of events leading to neuronal degeneration in PD. This makes PGRN a particularly intriguing therapeutic target, as PGRN is a potent regulator of inflammation, both in the periphery and the CNS. We were, therefore, interested to see whether elevating PGRN levels, via ND-602 treatment, would impact the microglial activation characteristically seen with MPTP. MPTP-induced microglial activation was assessed by staining free-floating sections with fluorescein-conjugated isolectin B_4_ (ILB4). There was a significant elevation in the number of cells labeling for ILB4 in both the SN_C_ and striatum (Figure 5). This induction of ILB4 staining by MPTP was significantly attenuated in the SN_C_ and striatum ipsilateral to ND-602 treatment. Thus, ND-602 appears to reduce the elevation in microglial expression characteristic of PD pathology.

### ND-602 does not Alter MPTP Metabolism

In the brain, MPTP is taken up by astrocytes, where it is metabolized into MPP^+^, its active metabolite [22]. It is MPP^+^ which enters dopaminergic neurons to exert its toxic effects. In order to confirm that ND-602 does not simply protect neurons by altering MPTP metabolism, we examined tissue levels of MPP^+^ by HPLC. Thus, four weeks following unilateral intranigral infusion of lentiviral vector, animals were administered daily injections of the parkinsonsism-inducing neurotoxin, MPTP HCl, for 5 days. Two hours following the final injection, animals were sacrificed by cervical dislocation and the brains removed. The SN was dissected for analysis of MPP^+^ content by liquid chromatography/mass spectrometry [23]. No differences in tissue concentration (ng/mg wet tissue weight) of MPP^+^ were observed (GFP/ipsilateral = 4.81236, ±0.37327; GFP/contralateral = 4.24065, ±0.40413; ND-602/ipsilateral = 4.06845, ±0.53879; ND-602/contralateral = 3.87286, ±0.48213) (TREATMENT main effect, F_1,15_ = 0.85949, *p* = 0.3696; HEMISPHERE main effect, F_1,16_ = 1.9946, *p* = 0.1797; TREATMENT × HEMISPHERE interaction effect, F_1,31_ = 0.06612, *p* = 0.8008). These data indicate that elevated PGRN expression does not alter MPTP metabolism in the SN_C_ and suggest that the induction of PGRN overexpression by ND-602 serves to protect nigrostriatal neurons from MPTP toxicity through other mechanisms.

## Discussion

The association of *PGRN* mutations with neurodegenerative disorders, most notably FTLD, has triggered a surge of research into the role of PGRN in the CNS. Although a picture is still forming, PGRN has proven to be a potent regulator of neuroinflammation and also acts as an autocrine neurotrophic factor, important for long-term neuronal survival. Thus, enhancing PGRN expression may strengthen the cells resistance to disease. Here, we have demonstrated a means of elevating PGRN expression in nigrostriatal neurons through viral vector delivery. When PGRN expression was elevated in the SN_C_, nigrostriatal neurons were protected from MPTP toxicity in mice, along with a preservation of striatal dopamine content and turnover. This nigrostriatal protection was accompanied by reductions in markers of MPTP-induced inflammation and apoptosis as well as a complete preservation of locomotor function. This very robust neuroprotection was observed even though not all nigral neurons were transfected. Overall, however, ND-602 treatment more than doubled measures of PGRN immonolabeling in the SNc over sham controls. As PGRN is a secreted factor [24], that acts extracellularly to activate cell survival signalling, it may be that not all nigral neurons are required to be transfected in order to be protected.

Although the mechanism of action for these neuroprotective effects has yet to be determined, PGRN has been associated with various CNS actions, which could be of therapeutic benefit in PD. Excitotoxicity is one mechanism of action known to contribute to the neurodegenerative process in SN_C_ neurons of PD [25]–[27] and its animal models [28]. In cultures of cortical neurons, PGRN has been shown to protect against glutamate toxicity [29], [30] and mice overexpressing PGRN show reduced infarct size and functional deficits in an ischemic model [30]. In light of its role in PD pathogenesis, modulation of excitotoxicity may be one means by which ND-602 protects neurons [31] against MPTP toxicity.

Another hallmark feature of PD pathogenesis is oxidative stress, which is also a key feature of the MPTP mouse model used in this study [32]. Inhibition of the mitochondrial electron transport chain results in excessive generation of reactive oxygen species leading to oxidative stress [33]. The phosphoinositide 3-kinases (PI_3_K)/AKT signalling pathway regulates fundamental cellular functions, including cell growth, proliferation, and cell cycle [34]. The PI_3_K/AKT pathway is also involved in the regulation of cellular apoptosis under oxidative stress [31], [35]–[37], making this pathway a good therapeutic target for oxidative stress-related neurodegenerative disease, such as PD. Indeed, this pathway has been implicated in the neuroprotective actions of rasagiline, a putative neuroprotectant for PD [38]. PGRN has been demonstrated to activate the PI_3_K/AKT pathway in primary neuronal cultures, resulting in protection against oxidative stresses triggered by H_2_O_2_ and MPP^+^ [29]. Conversely, depletion of PGRN renders primary neurons more vulnerable to both oxidative stress and excitotoxicity [39].

Oxidative stress in the CNS comes not only from mitochondrial-generated reactive oxygen species, but also from activated microglia [40]. PD patients have six times the number of reactive microglia of age-matched controls [41]. These reactive microglia are known to play a role in several neurodegenerative disorders, including PD [30], [42], [43] and similarly make a major contribution to MPTP-induced dopaminergic neurodegeneration [44]. Consistent with its robust expression in microglia, PGRN serves as a regulator of neuroinflammation [2], [13] through both antiinflammatory and proinflammatory processes. Macrophages deficient in PGRN showed decreased secretion of the anti-inflammatory cytokine interleukin-10, with a concomitant increase in the secretion of inflammatory cytokines such as interleukin-6 and TNF-α [11]. PGRN deficient mice display a dysregulated immune response in the brain, with a more pronounced age-dependent increase in glial activation [11], [45] and highly exaggerated inflammatory responses to various triggers, including LPS and bacterial infection [11]. Furthermore, microglia cultured from these mice were found to have toxic effects on co-cultured neurons [11]. Conversely, mice overexpressing PGRN display reduced pro-inflammatory cytokine release and elevated anti-inflammatory cytokine release in response to LPS [30]. Here, we report nigral and striatal microgliosis in response to MPTP in mice, a response significantly blunted by ND-602-induced elevations in PGRN. With inflammation playing such a significant role in PD pathogenesis and MPTP toxicity, the neuroprotective actions of ND-602, reported here, could result, at least in part, from regulation of inflammatory processes.

Neuroinflammatory mechanisms also contribute to the regulation of neural progenitor cell proliferation, possibly via Wnt signalling [25]. Wnt proteins are evolutionarily conserved secreted glycolipoproteins that play an important role in mediating cell proliferation, differentiation, cell fate determination during embryonic development and tissue homeostasis in the central nervous system in adult [27], [28], [32], [40]. Wnt signalling pathways may play a critical role in determining the balance between neuronal survival and death in degenerative disease [21], [46]. Wnt signalling also regulates the expression of other growth factors, such as BDNF [47], known to have trophic effects on dopaminergic neurons [48], [49] and to be reduced in PD patients [50], [51]. Accumulating evidence suggests that disruption in the Wnt signalling pathway may contribute to neurodegeneration in animal models of PD [22], [23], [26]. Indeed, mice deficient in PGRN show altered Wnt signalling, resulting in increased apoptosis [41]. This suggests a role for PGRN in regulating this signalling pathway. Mice deficient in PGRN also display impairments in neurite outgrowth [44], [52], neuronal arborization [43], [53], synaptic connectivity and plasticity [43]. These growth factor-like actions, mediated by activation of various cell growth/survival signalling pathways [29], [52], [54]–[56] may promote neuronal repair in response to injury or cellular stress. PGRN may also provide an injured cell with sufficient time for such self-repair by regulating the rate of apoptotic cell clearance [57], [58]. This PGRN-mediated shift in the dynamic equilibrium toward cell survival rather than death could contribute to the neuroprotective effects observed in this study.

Collectively, recent findings suggest that PGRN functions as a neuronal growth factor, supporting neuronal survival [20], [21], as well as a critical regulator of CNS inflammation [11], [15], [16]. Thus, PGRN has the potential to influence susceptibility to a wide range of neurodegenerative diseases, including motor neuron disease, lysosomal storage disease, Huntington’s disease, and Alzheimer’s disease [3], [17]–[19]. Mutations in *PGRN* were first identified in association with FTLD [3]–[5]. TAR DNA binding protein of 43 kDa (TDP-43) is the major pathological protein identified in the cellular inclusions in FTLD and plays a key role in forming proteinaceous aggregates. PGRN is a TDP-43 mRNA target and is required for appropriate proteolytic cleavage of TDP-43 [59]. Abnormal cleavage of TDP-43 can promote abnormal protein aggregation and cellular toxicity [60], features linked not only to FTLD, but also amyotrophic lateral sclerosis [61] and Huntington’s disease [62], [63]. Thus, in addition to promoting neuronal survival and regulating inflammatory responses, PGRN may also regulate protein misfolding, another shared feature of various neurodegenerative diseases.

In summary, the present study demonstrates that overexpression of PGRN in the SN_C_, by lentiviral delivery, can strengthen nigrostriatal resistance to MPTP toxicity in a mouse model of PD. Through its multiple actions as a neurotrophic factor and anti-inflammatory, PGRN impacts many of the events characteristic of PD pathophysiology, including oxidative stress, apoptosis, excitotoxicity, inflammation, and progenitor cell impairments. Thus, our results suggest that ND-602 may represent a promising new gene therapy for therapeutic intervention in PD and perhaps other neurodegenerative diseases sharing similar pathogenic hallmarks.
